# Involvement of cortical midline structures in the processing of autobiographical information

**DOI:** 10.7717/peerj.481

**Published:** 2014-07-22

**Authors:** Helder F. Araujo, Jonas Kaplan, Hanna Damasio, Antonio Damasio

**Affiliations:** 1 Brain and Creativity Institute, University of Southern California, Los Angeles, USA; 2 Neuroscience Graduate Program, University of Southern California, Los Angeles, USA; 3 Graduate Program in Areas of Basic and Applied Biology, University of Porto, Porto, Portugal

**Keywords:** Self, Autobiographical self, Cortical midline structures, Autobiographical memory, Medial prefrontal cortex, Posteromedial cortex

## Abstract

The term autobiographical self has been used to refer to a mental state that permits reflection on self-identity and personality and the answer to related questions (Damasio, 1998). It requires the retrieval and integrated assembly of memories of facts and events that define an individual’s biography. The neural mechanisms behind this state have not been fully elucidated, but it has been suggested that cortical midline structures (CMSs) are critically involved in processing self-related information. To date, the investigation of the involvement of CMSs in autobiographical-self processes has largely focused on the comparison between self and other in relation to one domain of information, personality traits, and has yielded conflicting results. Here, we investigated how activity in CMSs varies with (1) the target of the information (self versus an acquaintance), (2) the domain of information (personality traits versus facts), and (3) differences across individuals regarding how descriptive and how important/relevant the information targeted by the questions was, and regarding the amount of memory retrieved in order to answer the questions. We used an fMRI block-design in which 19 participants answered questions about traits and biographic facts, in relation to themselves and a distant acquaintance. In addition, the participants rated the descriptiveness and importance of the information targeted by the questions, and estimated the amount of memory retrieved to answer the questions. Our results showed that CMSs were active for both facts and traits and for both self and other, and that the level of activity in the posteromedial cortices was generally higher for other than for self. Moreover, the activity in CMSs also varied with the amount of memory retrieved to answer the questions and with descriptiveness and importance of the information. These findings suggest that involvement of CMSs during the evaluation of information is not specific for self, and depends on varied factors related to memory retrieval prompted by the questions and to processes required to answer them.

## Introduction

The term autobiographical self has been used to refer to a mental state that enables an individual to reflect on self-identity and personality, and to answer related questions (Damasio, 1998). This state requires retrieval and integrated assembly of memories of facts and events defining an individual’s biography. The neural mechanisms behind autobiographical-self processes have not been fully elucidated but it has been suggested that cortical midline structures (CMSs), particularly the medial prefrontal (MPFC), and posteromedial cortices (PMC), are critically involved in processing self-related information (Northoff & Bermpohl, 2004).

To date, the investigation of the involvement of CMSs in autobiographical-self processes has often focused on the comparison between “self” and “other” in relation to one domain of information, personality traits, and has yielded conflicting results as has been noted by others (Legrand & Ruby, 2009). For example, self-traits, contrasted with other-traits, revealed greater activity in the MPFC and PMC (e.g., D’Argembeau et al., 2007); but elsewhere, other-traits contrasted with self-traits yielded greater activity in these same regions (e.g., Pfeifer, Lieberman & Dapretto, 2007).

Interestingly, both the MPFC and PMC have been shown to consist of highly connected hubs (Tomasi & Volkow, 2011) and to be part of the so-called default network, yielding greater activity for internally oriented tasks than for externally oriented tasks (Buckner, Andrews-Hanna & Schacter, 2008). These regions do not appear to support processes specific to self but are rather able to assist varied processes involving internally generated representations. Moreover, we theorize that the involvement of CMSs during evaluative tasks used to investigate the autobiographical self relates to the processes that underlie those tasks, namely, memory retrieval prompted by the questions and the decisions needed to answer the questions, similarly to what has been proposed by other authors (Legrand & Ruby, 2009).

Accordingly, we hypothesize that the level of activity in CMSs during such tasks is likely to be commensurate with the level of processing related to memory retrieval (which depends, for example, on the effort in retrieving the memories and on the complexity of the retrieved memories), and to be related to decision making (which depends, for example, on the effort involved in the decision). Thus rather than being necessarily greater for self than for other, activity in CMSs may vary with: (1) the target of the evaluation (e.g., whether it pertains to self, to a close other, or to a distant other); (2) the domain of information being evaluated (e.g., personality traits or demographic facts); and (3) differences across participants regarding the information they have to evaluate (e.g., differences in relation to how well the information describes self and other, and to the importance the information has to the participants’ image of self or of other).

The above factors have already been shown to contribute to differences in behavioral tasks (e.g., response times to the questions) between self and other (Symons & Johnson, 1997), but have only been partly investigated in neuroimaging studies. Specifically, it has been established that brain activity during the evaluation of traits depends on the target of that evaluation. For example, the difference of activity in the MPFC between self and other has been shown to depend on who the other is in relation to self (i.e., whether it is a close or a distant other) (Denny et al., 2012). The contribution of the other factors, however, needs further investigation.

In this study, we tested the above set of hypotheses and investigated how brain activity varies according to the factors listed above. To do so we conducted an fMRI block-design study, in which participants answered questions about biographical information. The questions varied in terms of the target of evaluation and the domain of information being evaluated. In addition, differences across participants in relation to the information being evaluated were measured after scanning. We used an active baseline (the one-back task) to obtain a strong contrast between the experimental conditions and the baseline. We expected this baseline task to suspend the introspective processes likely to occur during rest or poorly engaging tasks, and to deactivate CMSs, given that CMSs are known to be especially active during rest (as reviewed in Buckner, Andrews-Hanna & Schacter, 2008).

The target of the evaluation in our study was the participant (self) or a distant acquaintance of the participant (other). This choice of other was made in the hope of attaining a clear distinction between self and other, and is, as explained above, likely to be a decisive factor in the differences of brain activity between self and other. Memories for a distant acquaintance are probably less numerous, less frequently retrieved, and less readily accessible than autobiographical memories. In addition, it is likely that individuals are more likely to abstract summary representations for information pertaining to themselves or close acquaintances than for information pertaining to distant acquaintances (Fuhrman & Funder, 1995). Accordingly, we expected that in our study, compared with evaluating other, evaluating self should require a lower level of processing memory retrieval and decision-making and thus should yield a lower level of related activity in CMSs.

**Figure 1:**
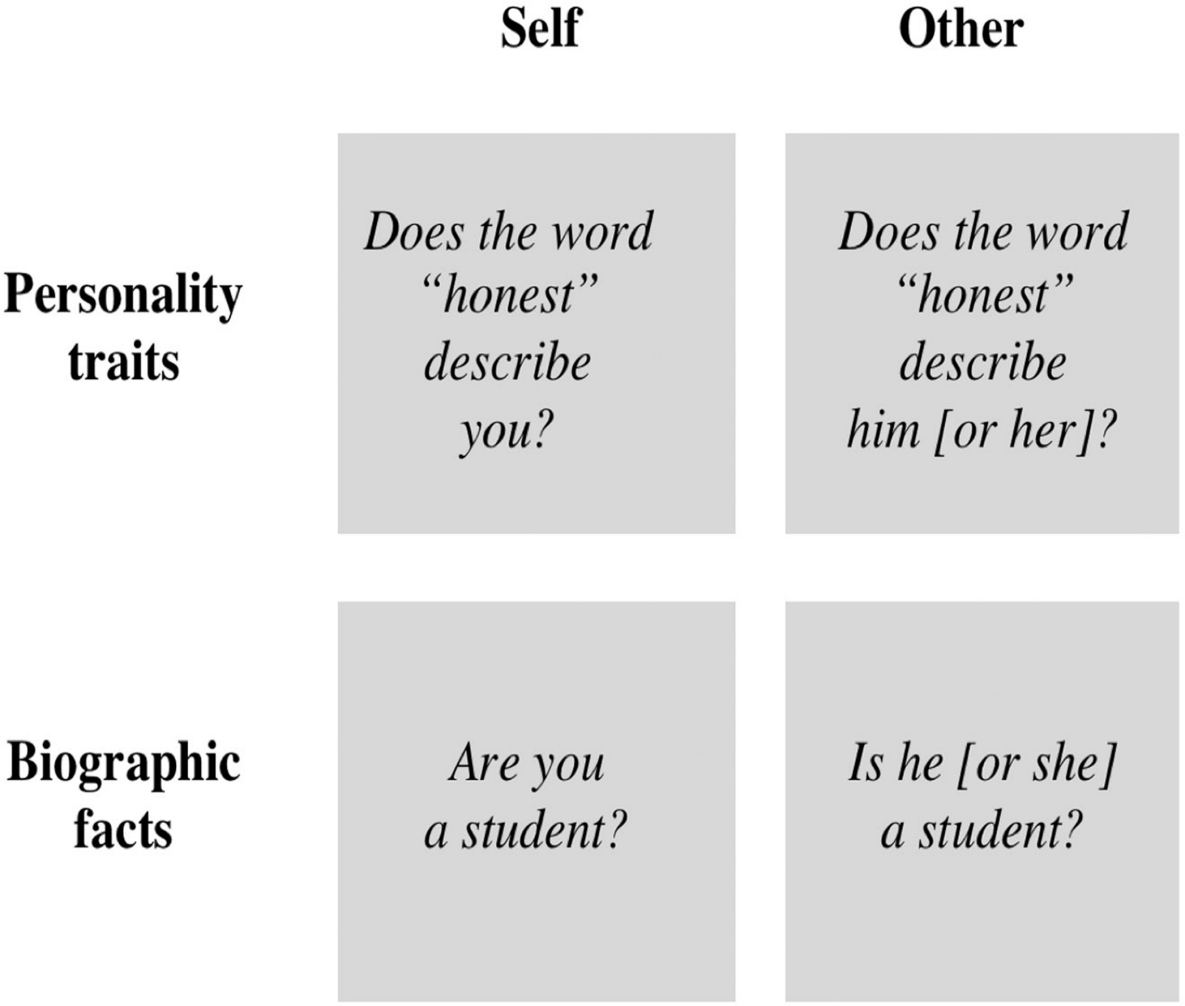
Experimental conditions used in the fMRI study. The conditions varied according to the target of the evaluation (self versus other) and to the domain of information evaluated (personality traits versus biographic facts).

The study included two domains of information: personality traits and basic facts that define one’s identity (e.g., age, height, nationality and occupation), to which we refer as “biographic facts” (Fig. 1). Although both of these information domains are part of the general knowledge a person is likely to hold regarding one self (Klein & Gangi, 2010), biographic facts are significantly different from personality traits (Keenan, Golding & Brown, 1992). Specifically, biographic facts are objective, indisputable and easily verifiable (e.g., the personal information contained in one’s drivers license or identity card). On the other hand, personality traits (e.g., honesty) are classified in terms of terms of valence and desirability (Anderson, 1968) and are generally associated with an emotional significance (Klein & Loftus, 1993). Moreover, the judgment of our personality traits depends on our life experience. The number of daily experiences that provide knowledge regarding certain personality traits may be very limited and thus one’s representations for those traits may remain relatively ambiguous (Kuiper, 1981). In brief, compared with facts, traits are less objective and less easily verifiable and hold a greater emotional significance. Thus the processes of memory retrieval and decision-making pertaining to the evaluation of traits are likely to evoke greater emotional responses and related brain activity than those processes pertaining to the evaluation of facts.

We assessed differences regarding how biographical information was processed by the participants, by using three measures: descriptiveness (i.e., the ratings of how well the content of the questions described self or other); importance (i.e., how important the content of the questions was to address the participants’ self-image or the participants’ image of their acquaintances); and amount of memory retrieved to answer the questions (i.e., the participants’ estimates of number of episodes retrieved to answer the questions) (Table 1).

**Table 1:** Participants’ ratings and estimates collected after scanning. The participants rated the information targeted by each questions in terms of descriptiveness and importance, and estimated the amount of memories they needed to retrieve to answer the questions.

	Self	Other
Descriptiveness	How well does the information targeted by each question describe “self”?	How well does the information targeted by each question describe “other”?
Importance	How important to the participants’ self-image was the information targeted by each question?	How important to the participants’ image of their acquaintance was the information targeted by each question?
Memory retrieved to answer the questions	How many memories (episodes) did the participants need to retrieve in order to answer the questions in each condition?	

Descriptiveness has been shown to relate to participants’ response times to the questions about self (e.g., Markus, 1977). Specifically, it has been shown that the response times to self-trait questions are shorter for traits considered highly descriptive or highly non-descriptive (i.e., unambivalent) traits, than for traits that are considered intermediately descriptive (ambivalent) traits. Furthermore, this difference of processing time may occur because individuals are more likely to hold unambivalent cognitive generalizations (self-schemata, Markus, 1977) or summary representations (Klein & Loftus, 1993) for highly descriptive or highly non-descriptive traits than for intermediately descriptive traits. For these reasons, we believe that the level of CMSs activity should be lower for individuals that considered the information highly descriptive or highly non-descriptive because these individuals are likely to require lower level of processing related to memory retrieval and decision making to answer the questions. To test this, we assessed the participants’ ratings for descriptiveness and used those ratings to compute a measure of how ambivalent the information was considered. Specifically, *descriptive ambivalence* scores ranged from 0 (information that the participants rated as highly descriptive or highly non-descriptive) to 2 (information that the participants rated as equally descriptive and non-descriptive).

In addition, the importance of biographical information has also been shown to be relevant in understanding behavioral measures of processing information (Markus, 1977). We used this measure to complement the information provided by the descriptiveness. An individual’s importance rating for a biographic datum (independently of its descriptiveness) probably reflects the likelihood of having given prior consideration to that information, and thus the likelihood of having a summary representation for it. Consequently, the level of CMSs activity should also be lower for individuals who considered the information more important because these individuals probably required lower level of processing related to memory retrieval and decision making to answer the questions.

The participants’ estimates of number of episodes retrieved to answer the questions were used to assess how much retrieval of specific events or episodes each condition required. Behavioral studies have shown that judging personality traits for both self and other may rely on relatively more effortful process of retrieving of specific episodes (exemplars) or on a relatively less effortful process of retrieving summary representations (Klein & Loftus, 1993). Accordingly, we expected that level of CMSs activity should be higher for individuals who estimated greater amount of memory retrieved to answer the questions because these individuals probably required higher level of processing related to memory retrieval and decision-making.

## Methods

### Participants

Twenty native English speakers, right-handed, with no history of neurological diseases, were recruited from the University of Southern California community. The participants were paid for their participation, and provided written informed consent following the Institutional and Federal Guidelines. Data from one participant had to be excluded due to motion artifacts. The final sample comprised 19 participants (10 female, and 9 male; 20.1 ± 1.3 years old).

### Materials and procedures

Before scanning, participants were asked to select an acquaintance of the same gender, whom they knew well enough to answer questions about his/her personality and life in general, but with whom they did not have a strong emotional connection (similar criteria to those used by Modinos, Ormel & Aleman (2009)). Mean age of the acquaintances was 20 (*SD* = 1.2) years old, and mean duration of acquaintanceship was 25.8 (*SD* = 27.95) months.

The stimuli were questions organized in four conditions (Fig. 1): (i) self-traits, in which the participants were asked to judge if specific personality traits described them accurately (e.g., “Does the word honest describe you?”); (ii) self-facts, in which participants were asked to verify if specific autobiographical facts were correct (e.g., “Are you a student?”); (iii) other-traits, in which the participants were asked to judge if specific personality traits described their acquaintances properly (e.g., “Does the word honest describe him [or her]?”); (iv) other-facts, in which the participants were asked to verify if specific biographical facts about their acquaintances were correct (e.g., “Is he [or she] a student?”). The content of the questions was the same for self and for other (Table S1). The trait-questions comprised 27 negative traits and 27 positive traits, and derived from a published list of personality traits rated for likableness and meaningfulness (Anderson, 1968). Negative traits were selected among the most disliked adjectives from that list, and positive traits were selected among the most liked adjectives from the list; in both cases, only the traits with the highest meaningfulness scores were selected. The fact-questions covered general aspects of one’s life, such as age, height, weight, ethnicity, nationality, occupation, typical means of transportation, household and physical appearance. The participants had three options to answer the questions: “yes”; “no”; or other (when the participants did not know the correct answer, or did not consider “yes” or “no” as the correct answer).

We used MATLAB (The Mathworks) and Psychophysics Toolbox Version 3 (Brainard, 1997) to present the stimuli (projected on a screen, which the participants saw through a mirror mounted on the head coil) and to record the participants’ responses (given with button presses using the right hand).

The fMRI experiment followed a block design. Each run contained three blocks for each condition. The order of the blocks was randomized in each run and for each participant. Each block lasted 24 s and contained a random selection of six questions. Each question was presented for four seconds, during which participants were asked to provide the requested answer. Blocks of stimuli were separated by a 24-s block of the one-back-task baseline, in which the participants saw a series of letters and had to decide whether each of the letters was identical to the one immediately preceding it.

### Image acquisition

Magnetic resonance images were acquired with a 3-Tesla Siemens MAGNETON Trio System. We used the following parameters for echo-planar image (EPI) acquisition: TR = 2000 ms, TE = 25 ms, flip angle = 90°, 64 × 64 matrix, in-plane resolution 3.0 mm × 3.0 mm, 41 transverse slices, each 3 mm thick, and field of view covering the whole brain. Each functional run lasted 9.7 min and consisted of 291 volumes. All participants completed three runs, but due to a problem with the software, one participant did not complete two blocks (one for other-traits, and one for other-facts); the EPI volumes corresponding to these blocks were not included in the analysis. For purposes of registration, we also acquired structural images (T1-weighted magnetization-prepared rapid gradient echo, MPRAGE) using the following parameters: TR = 1950 ms, TE = 2.3 ms, flip angle = 7°, 256 × 256 matrix, 193 coronal slices, 1 mm isotropic resolution.

### Questionnaires and analysis

After the scanning, the participants estimated the amount of memory (e.g., particular episodes) they recalled in order to answer the questions in each condition, using a 7-point Likert scale (1: *I did not recall any particular memory.;* 7: *I recalled many episodes…*). The participants also rated the content of each self-question in terms of how well it described them (self-descriptiveness) and of how important (self-importance) it was to their identities; likewise, for the content of each other-question relative to how well it described their acquaintances (other-descriptiveness) and how important it was to their representations of the acquaintances (other-importance). For these ratings, each question was transformed into a statement, (“*You are a student”.*), and Likert scales were used. The scales ranged as follows: (i) for self-descriptiveness, from 1 *(…definitely untrue or uncharacteristic of me.*) to 5 (*…very true or strongly characteristic of me.);* (ii) for self-importance, from 1 (*…definitely not important to my identity*.) to 5 (*…absolutely important to my identity.*); (iii) for other-descriptiveness, from 1 (*…definitely untrue or uncharacteristic of him /her*.) to 5 (*…very true or strongly characteristic of him /her*.); (iv) for other –importance, from 1 (*…definitely not important to the image I have of him/her*.) to 5 (*…absolutely important to the image I have of him/her*). The ratings of descriptiveness were recoded in order to assess how ambivalent (descriptive ambivalence) the participants considered the questions, as follows: ratings 5 and 1 were recoded into 0, ratings 4 and 2 were recoded into 1, and rating 3 was recoded into 2. All behavioral data were analyzed using repeated measures ANOVA, and Pearson correlation, with PASW Statistics 18.0.

### Image processing and analysis

The functional data were preprocessed and analyzed with FSL (FMRIB’s Software Library, www.fmrib.ox.ac.uk/fsl). Before the statistical analysis was performed, the following steps were applied: (i) motion correction using MCFLIRT (Jenkinson et al., 2002); (ii) slice-timing correction using Fourier-space time-series phase-shifting; (iii) non-brain removal using BET (Smith, 2002); (iv) spatial smoothing using a Gaussian kernel of FWHM 5 mm; (v) grand-mean intensity normalization of the entire 4D dataset by a single multiplicative factor; (vi) high-pass temporal filtering (cutoff period = 100 s; gaussian-weighted least-squares straight line fitting, with sigma = 50.0 s). The participants’ data were registered to their high-resolution structural images (with 7 degrees of freedom) and to a standard space (MNI-152 atlas, with 12 degrees of freedom) using the FMRIB’s Linear Image Registration Tool (Jenkinson et al., 2002) and FNIRT nonlinear registration (Andersson, Jenkinson & Smith, 2007). The data were first analyzed using FSL’s implementation of the general linear model, FEAT Version 5.98. The model included motion parameters, and four separate regressors, one for each experimental condition. Each regressor was derived from the convolution of the corresponding task design and a gamma function representing the hemodynamic response. Time-series statistical analysis was carried out using FILM with local autocorrelation correction (Woolrich et al., 2001). The functional data of the three runs for each participant were combined using a fixed-effect model, which forces the random effects variance to zero in FLAME (FMRIB’s Local Analysis of Mixed Effects; Woolrich et al., 2004). The data from all the participants were then analyzed in a higher-level mixed-effects analysis using FLAME. Two additional analyses included behavioral measures as inter-individual factors (covariates) in this last step: (i) the participants’ mean descriptive ambivalence scores and participants’ mean importance scores; (ii) the participants’ estimates of the amount of memory they recalled. We applied the following threshold to all statistical images: clusters determined by *Z* > 2.3 and a corrected cluster size significance threshold of *p* = .05 (Worsley, 2001). In addition, we performed a conjunction analysis of the higher-level results for each condition compared with the baseline, using easythresh_conj script in FSL (Nichols et al., 2005); for this analysis, we used the whole-brain as a mask and a threshold of *Z* > 2.3, *p* < .05.

## Results

### Behavioral data

#### Response time

Questions about self were answered faster than those about other, *F*(1, 18) = 31.42, *p* < .0001. In addition, questions about facts were answered faster than those about personality traits, but this difference did not reach the level of statistical significance, *F*(1, 18) = 4.03, *p* = .60. A statistically significant interaction between the target of the evaluation (self or other) and the domain of information targeted by the questions (facts or traits) was observed, *F*(1, 18) = 10.17, *p* < .005. Pairwise comparisons (*p* values adjusted for multiple comparisons using Bonferroni correction) revealed that differences of response times between self and other were statistically significant for facts (*p* < .0001) but did not reach statistical level of significance for traits (*p* < .24); in addition, differences of response times between facts and traits were statistically significant for self (*p* < .016) but not for other (*p* < 2.704) (Table 2).

**Table 2:** Mean response time, descriptive ambivalence scores (ranging from 0 to 2), importance ratings (ranging from 1 to 5), and memory estimates (ranging from 1 to 5) for facts and traits relative to self and to other.

	Response time		Descriptive ambivalence		Importance		Memory	
Condition	*M*	*SD*	*M*	*SD*	*M*	*SD*	*M*	*SD*
**Self**								
**Facts**	1.37	.22	0.17	.03	2.49	.67	2.8	1.6
**Traits**	1.51	.27	0.59	.06	3.28	.01	3.4	1.8
**Other**								
**Facts**	1.57	.21	0.26	.05	2.14	.70	4.5	1.5
**Traits**	1.58	.30	0.68	.08	3.12	.92	4.6	1.5

#### Response time according to the valence of the traits

We did not observe a statistically difference of response times between negative traits and positive traits, for self or for other (Table 3).

**Table 3:** Mean response time (seconds), descriptiveness ratings (1–5), and importance ratings (1–5) for negative and for positive traits regarding self and other.

	Response time		Descriptiveness		Importance	
Traits	*M*	SEM	*M*	*SEM*	*M*	SEM
**Self**						
**Negative**	1.50	0.06	1.62	.39	2.73	1.41
**Positive**	1.51	0.07	4.12	.37	3.83	.70
**Other**						
**Negative**	1.52	0.08	1.77	.76	1.77	1.26
**Positive**	1.65	0.07	3.64	.61	3.43	.78

#### Response time according to descriptiveness ratings

**Figure 2:**
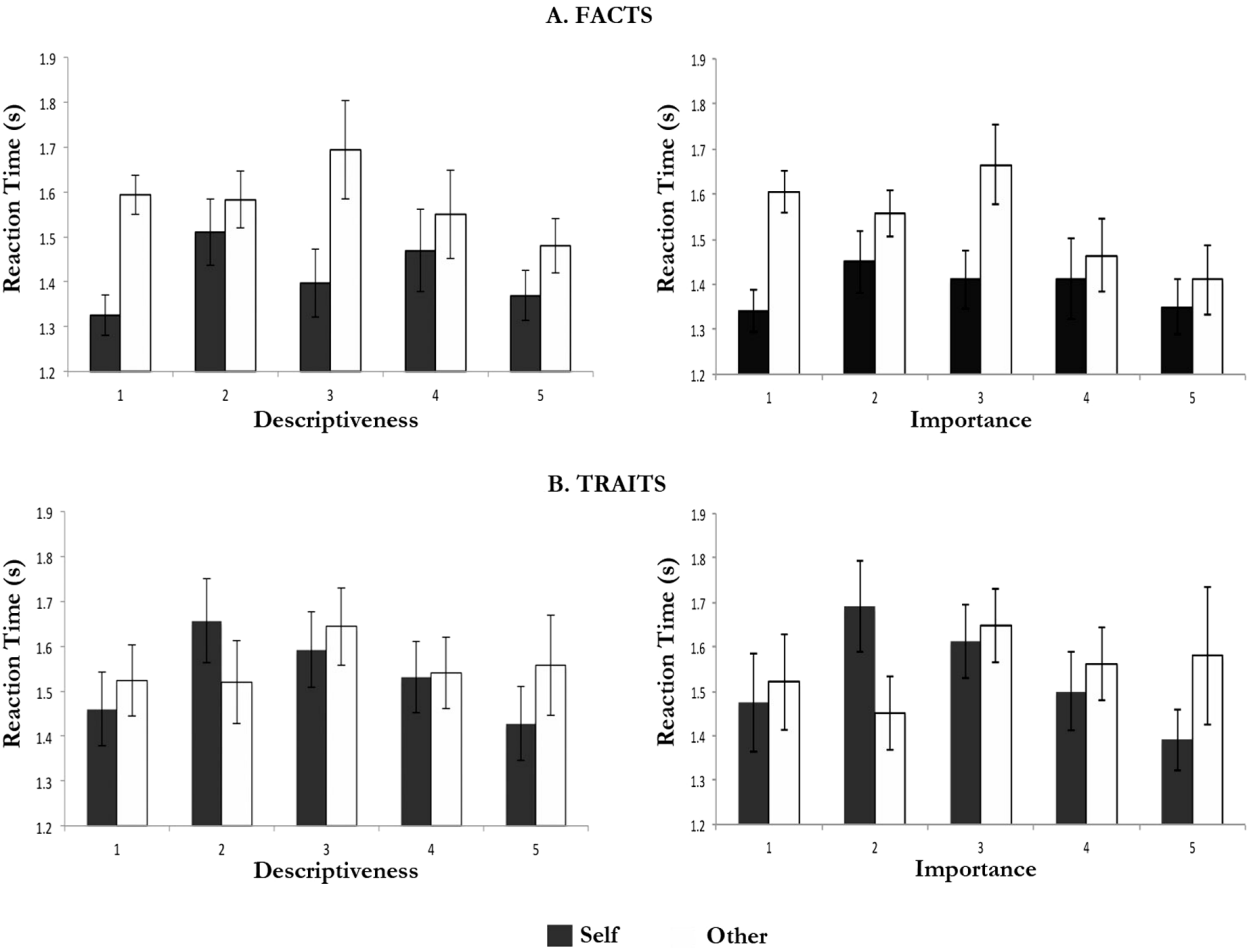
Response times (*M* and *SEM*) according to descriptiveness and importance ratings.

Facts rated 1 or 5 (extremes) in terms of descriptiveness showed shorter response times than facts rated intermediate values of descriptiveness (Fig. 2). These differences reached statistical significance for the comparison between self-facts rated 1 for descriptiveness and self-facts rated 2 for descriptiveness, *F*(1, 17) = 12.075, *p* < .003; and that between self-facts rated 5 for descriptiveness and self-facts rated 2 for descriptiveness, *F*(1, 17) = 5.381, *p* < .033. In addition, response times for other-facts rated 5 for descriptiveness were also shorter than those for other-facts rated 3 for descriptiveness *F*(1, 17) = 5.424, *p* < .032.

Traits showed a similar trend (Fig. 2). These differences reached only statistically significance for the comparison between self-traits rated 1 for descriptiveness and self-traits rated 2 for descriptiveness, *F*(1, 18) = 9.440, *p* < .007; and that between self-traits rated 5 for descriptiveness and self-traits rated 2 for descriptiveness, *F*(1, 17) = 6.855, *p* < .018.

#### Response time according to importance ratings

The comparison of mean response times according to the importance ratings was limited by the fact that not all participants used the full range of the importance ratings (e.g., only 12 participants rated any self-traits 1 for importance). Nonetheless, the analysis revealed similar trends to those described above for descriptiveness.

Facts rated 1 or 5 (extremes of importance) showed shorter response times than facts rated intermediate importance values (Fig. 2). These differences were statistically significant for the comparison between self-facts rated 1 for importance and self-facts rated 2 in the same regard, *F*(1, 17) = 6.125, *p* < .048; and for the comparison between other-facts rated 5 for importance and (i) other-facts rated 2 for importance, *F*(1, 12) = 7.131, *p* < .020, and (ii) other-facts rated 3 for importance, *F*(1, 12) = 5.622, *p* < .035. On the other hand, for other, facts rated 1 for importance were associated with longer response times than facts rated 5 for importance, *F*(1, 12) = 6.287, *p* < .028.

Traits rated 1 or 5 (extremes) in terms of importance were also associated with shorter response times than traits rated intermediate values of importance (Fig. 2). These differences reached only statistically significance for the comparison between self-traits rated 5 for importance and (i) self-traits rated 2 for importance, *F*(1, 9) = 6.125, *p* < .035, and (ii) self-traits rated 3 for importance, *F*(1, 12) = 6.391, *p* < .027.

#### Descriptiveness

Both for facts and for traits, information targeted by the questions was considered less ambivalent (i.e., descriptiveness ratings were closer to highly descriptive or highly non-descriptive) for self than for other, *F*(1, 18) = 4.619, *p* < .045 (Table 2). Facts were also considered less ambivalent than traits, *F*(1, 18) = 54.16, *p* < .0001 (Table 2). No statistically significant interaction between the target of the evaluation (self or other) and the domain of information targeted by the questions (facts or traits) was observed for descriptiveness ambivalence scores.

In addition, descriptiveness ratings were greater for positive traits than for negative traits, for both self and other, *F*(1, 18) = 168.752, *p* < .0001 (Table 3). A statistically significant interaction between the target of the evaluation (self or other) and the valence of the traits (negative or positive) was observed for descriptiveness ratings, *F*(1, 18) = 4.855, *p* < .041. Pairwise comparisons (*p* values adjusted for multiple comparisons using Bonferroni correction) showed that descriptiveness ratings were greater for positive traits than for negative traits, both for self (*p* < .0001), and for other (*p* < .0001); in addition, descriptiveness ratings were greater for self than for other in relation to positive traits (*p* < .008) but not in relation to negative traits (*p* < 1.584).

#### Importance

Participants’ ratings of importance were greater for self than for other, *F*(1, 18) = 8.85, *p* < .008. Furthermore, for both self and other, ratings of importance were greater for traits than for facts, *F*(1, 18) = 31.27, *p* < .001. A statistically significant interaction between the target of the evaluation (self or other) and the domain of information targeted by the questions (facts or traits) was observed, *F*(1, 18) = 4.431, *p* < .05. Pairwise comparisons (*p* values adjusted for multiple comparisons using Bonferroni correction) revealed that participants importance ratings were greater for traits than for facts, both for self (*p* < .0001) and for other (*p* < .001); in addition, participants importance ratings were greater for self than for other in relation to facts (*p* < .004) but not in relation to traits (*p* < .504).

Positive traits were considered more important than negative traits for both self and other (Table 3) *F*(1, 18) = 18.064, *p* < .0001, but a statistically significant interaction between the target of the evaluation (self or other) and the valence of the traits (negative or positive) was found for importance ratings, *F*(1, 18) = 8.16, *p* < .010. Pairwise comparisons (*p* values adjusted for multiple comparisons using Bonferroni correction) showed that participants’ importance ratings were greater for positive traits than for negative traits, both for self (*p* < .0001) and for other (*p* < .044); in addition, participants’ importance ratings were greater for self than for other in relation to positive traits (*p* < .016) but not in relation to negative traits (*p* < 2.444).

#### Memory retrieved to answer the questions

Participants’ estimates of memory retrieved to answer the questions were higher for other than for self, both for biographic facts and for traits, *F*(1, 18) = 32.02; *p* < .001 (Table 2).

#### Correlation between behavioral measures

Mean response time for other-facts correlated negatively with mean importance ratings for other-facts, *r*(18) = −.548, *p* < .015. No other statistically significant correlations were found for the measures reported above.

### Functional imaging data

#### Task versus baseline (one-back-task)

**Figure 3:**
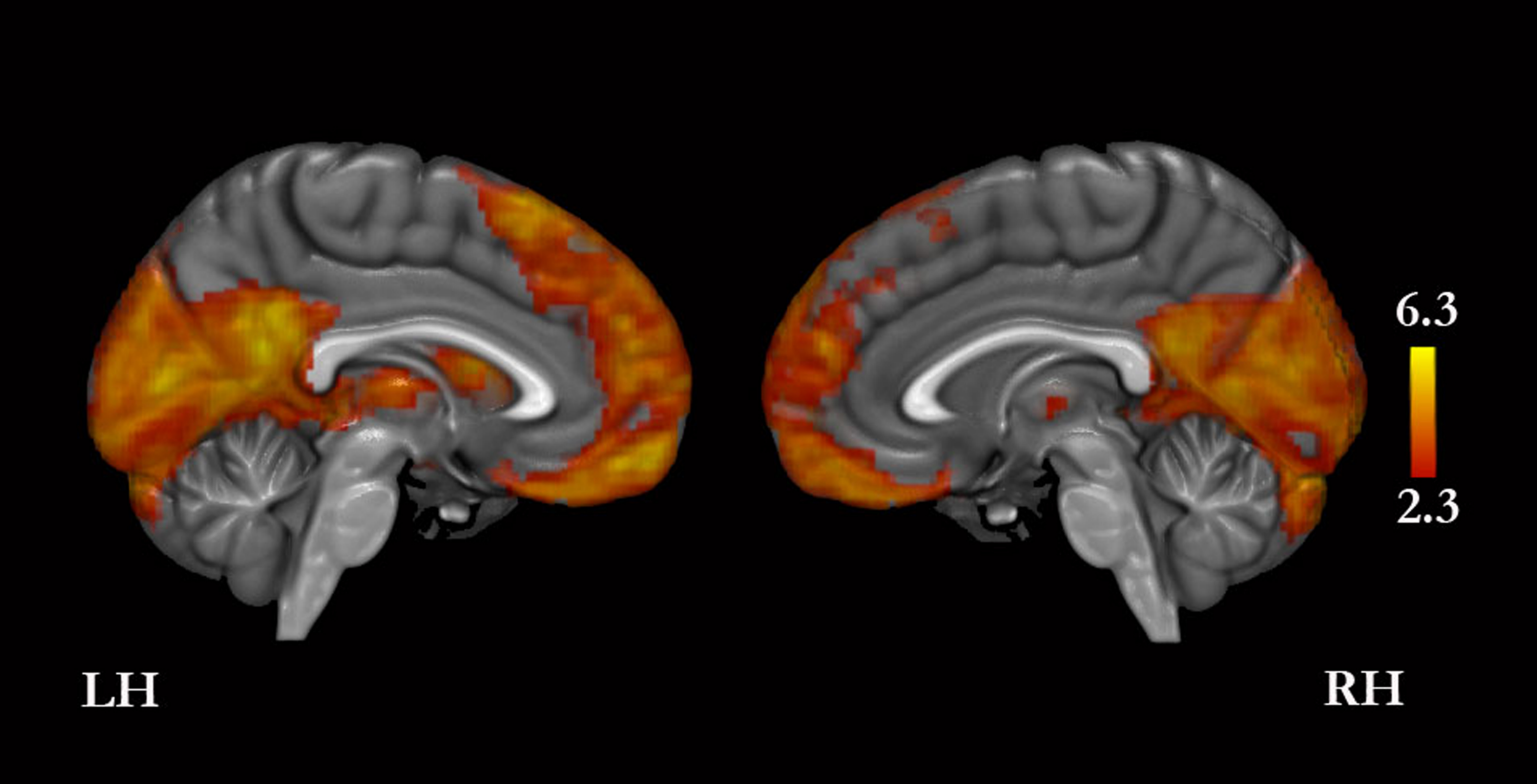
Conjunction analysis for the experimental conditions versus baseline. Only medial surfaces are shown. See Results and Table S1 for the remaining whole-brain results.

A conjunction analysis revealed that the four conditions compared with baseline yielded greater signal in, bilaterally, the MPFC, PMC, cuneus, orbitofrontal cortex, basal forebrain, inferior frontal gyrus, middle temporal gyrus, temporal pole, hippocampus, cerebellum, and thalamus, and in the left middle and superior frontal gyri, lateral occipital cortex, angular gyrus, amygdala, and caudate (Fig. 3 and Table S1).

#### Self versus other

**Figure 4:**
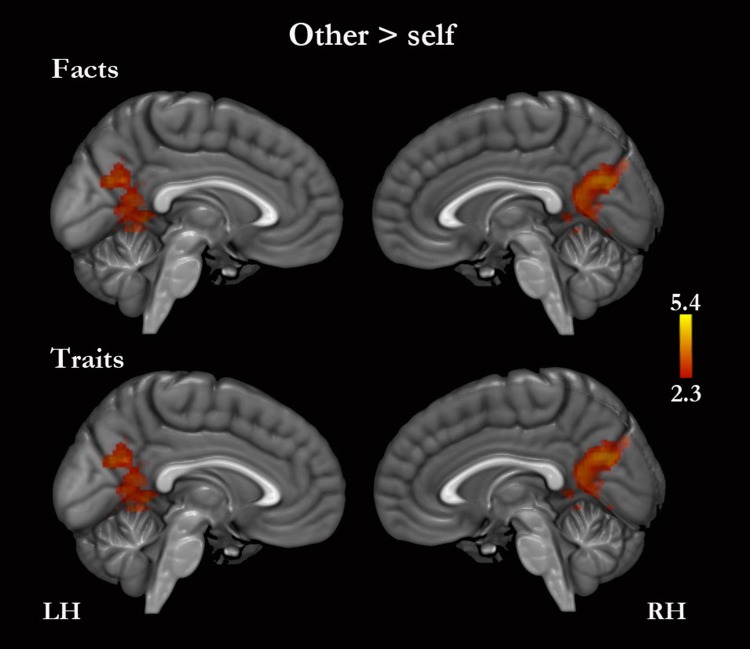
Other versus self. Only medial surfaces are shown. See Results and Table S2 for the remaining whole-brain results.

No regions of greater activity for self, compared with other, were detected for traits or for traits combined with facts. However, self-facts compared with other-facts were associated with greater activity in the right middle temporal gyrus, in the left postcentral gyrus, and bilaterally in the superior temporal, supramarginal and angular gyri (Table S2). Other compared with self was associated with greater activity in the PMC for traits and facts combined (Fig. 4, Table 4 and Table S2). Other-traits compared with self-traits were associated with greater activity in the left lateral occipital cortex, and bilaterally in the PMC (Fig. 4, Table 4 and Table S2).

**Table 4:** Summary of the results for the main contrasts in CMSs. Coordinates (*x*, *y*, *z*; MNI-152 standard space) and *Z*-scores correspond to the activation peaks (clusters *Z* > 2.3; cluster probability *p* < .05) in the MPFC and PMC for each of the main contrasts. (See Tables S2–S4 for the remaining whole-brain results.)

	**MPFC**					**PMC**				
	H	*x*	*y*	*z*	*Z*	H	*x*	*y*	*z*	*Z*
**Target of evaluation**										
Self > other	–					–				
Other > self	–					L	−8	−48	2	4.77
						R	6	−56	18	5.8
**Domain of information**										
Facts > traits										
Self	L	−10	26	−16	4.92	L	−4	−64	20	6.04
	R	8	30	−18	5.06	R	2	−58	20	5.98
Other	L	−8	32	−16	4.3	L	−4	−60	16	5.79
	R	10	36	−18	3.88	R	2	−54	12	5.01
Traits > facts										
Self	–					–				
Other	–					–				
**Target of evaluation and** **domain of information**										
Self > other										
Facts	–					–				
Traits	–					–				
Other > self										
Facts	–					L	−8	−48	0	3.62
						R	4	−54	18	3.78
Traits	–					L	−2	−68	22	4.59
						R	6	−56	18	5.36

#### Facts versus traits

**Figure 5:**
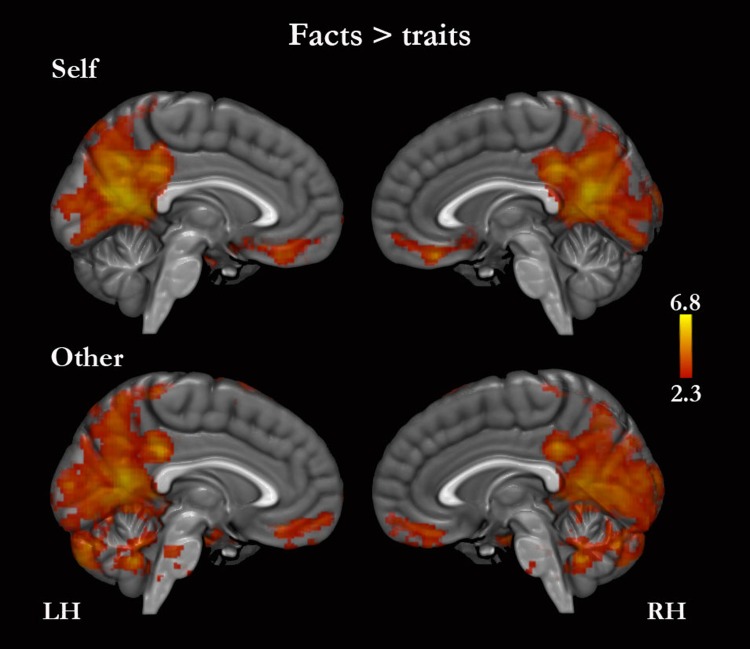
Facts versus traits. Only medial surfaces are shown. See Results and Tables S3 and S4 for the remaining whole-brain results.

**Figure 6:**
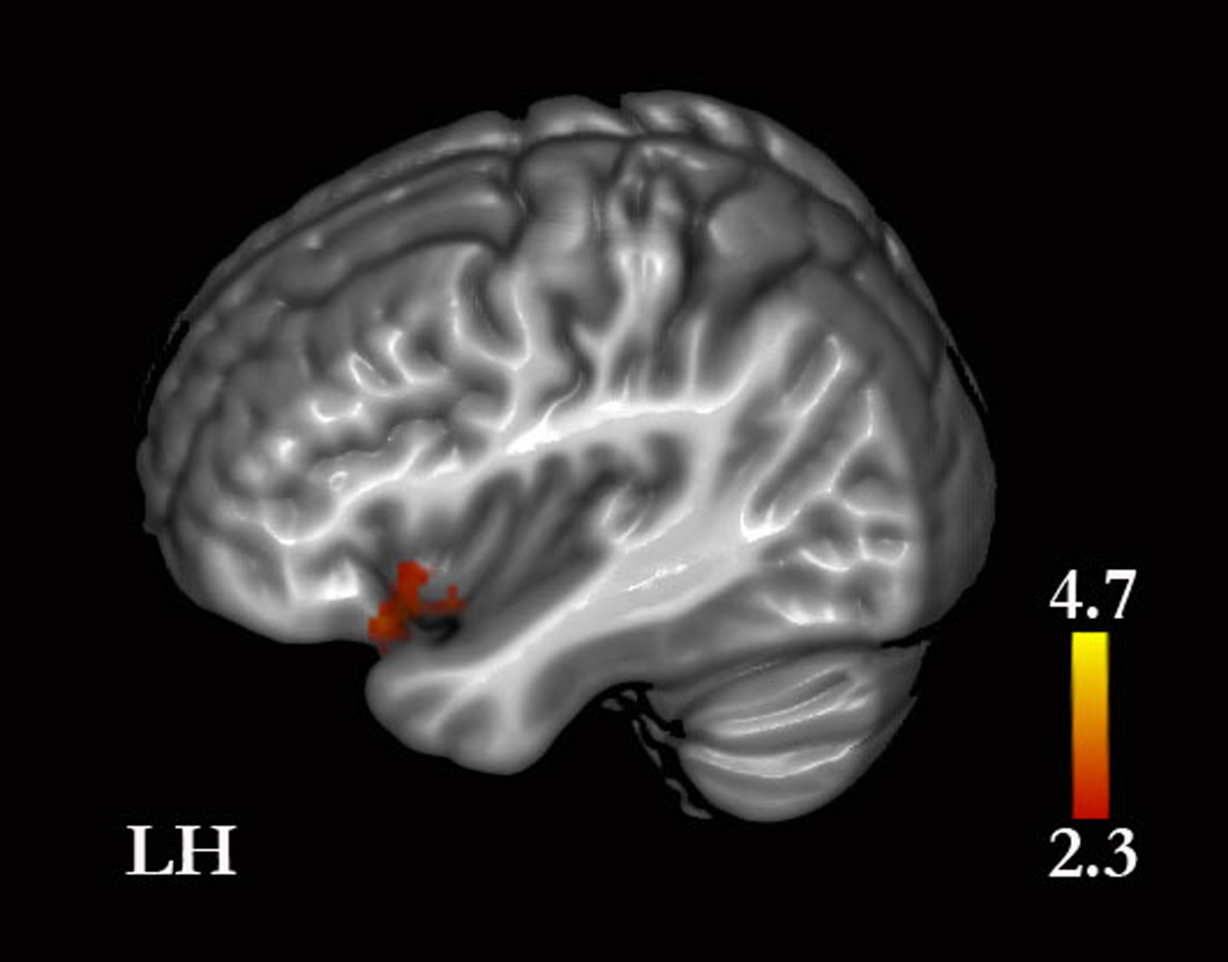
Self-traits versus self-facts. Greater signal was found in the insula and inferior frontal gyrus. (See Results and Table S3 for the remaining whole-brain results).

Facts (self-facts and other-facts combined) compared with traits (self-traits and other-traits combined) yielded greater activity in the left amygdala, caudate, basal forebrain, pons and medulla, and bilaterally in MPFC, PMC, middle frontal gyrus, precentral gyrus, middle and inferior temporal gyri, angular gyrus, lateral occipital, fusiform gyrus, hippocampus, cerebellum, thalamus, and mesencephalon. When self and other were analyzed separately, we found the following: (i) self-facts, compared with self-traits, were associated with greater activity in the left supramarginal gyrus and amygdala, and bilaterally in the MPFC, PMC, middle frontal gyrus, precentral gyrus, middle and inferior temporal gyri, angular gyrus, lateral occipital cortex, fusiform gyrus, hippocampus, and basal forebrain (Fig. 5, Table 4 and Table S3); (ii) self-traits, compared with self-facts, were associated with greater activity in the left insula, inferior frontal gyrus and orbitofrontal cortex, and in the right lateral occipital cortex (Fig. 5, Fig. 6 and Table S3); (iii) other-facts, compared with other-traits, were associated with greater activity in the left amygdala, supramarginal gyrus, thalamus, pons and medulla, and bilaterally in the MPFC, PMC, cuneus, middle frontal gyrus, precentral gyrus, middle and inferior temporal gyri, angular gyri, lateral occipital cortex, fusiform gyrus, hippocampus, and cerebellum (Fig. 5, Table 4 and Table S4); (iv) other-traits, compared with other-facts, did not show any statistically significant differences.

### Behavioral covariate analysis

#### Descriptive ambivalence, and importance

For each condition, the participants’ mean scores for ambivalence (based on the descriptiveness ratings) and the participants’ mean importance ratings were explored simultaneously as between-subjects factors.

Positive correlations between brain signal and participants’ mean *descriptive ambivalence* scores were found for three conditions (Table 5, Table S5 and Table S6): (i) for self-facts, bilaterally in the MPFC, anterior cingulate cortex, superior and middle frontal gyri, and cerebellum; (ii) for self-traits, bilaterally in the paracentral gyrus, posteromedial cortex, precentral and postcentral gyri, superior parietal lobule and pons, and in the right middle frontal gyrus and left mesencephalon; (iii) for other-traits, bilaterally in the medial prefrontal cortex, posteromedial cortex, and cerebellum, in the right frontal pole and orbitofrontal cortex, and in left postcentral gyrus/ superior parietal lobule.

**Table 5:** Activity in CMSs **positively** correlated with participants’ descriptive ambivalence, and estimates of amount of memory retrieved to answer the questions. Coordinates (*x*, *y*, *z*; MNI-152 standard space) and *Z*-scores correspond to the activation peaks (clusters *Z* > 2.3; cluster probability *p* < .05) in the MPFC and PMC. (See Tables S7 for the remaining whole-brain results.)

	**MPFC**					**PMC**				
	H	*x*	*y*	*z*	*Z*	H	*x*	*y*	*z*	*Z*
**Descriptive ambivalence**										
**Facts**										
Self	L	−18	40	30	3.35					
	R	14	38	40	3.79					
	L	−6	48	6	3.11					
	R	2	50	6	3.24					
Other	–									
**Traits**										
Self	–					L	−4	−78	46	3.47
						R	6	−52	62	2.8
Other	L	−2	32	−20	2.66	L	−8	−70	54	3.17
	R	2	32	−24	3.95	R	14	−74	56	3.86
**Memory**										
**Facts**										
Self	–					L	−6	−48	54	3.35
Other	–					L	−4	−46	60	3.31
**Traits**										
Self	L	−10	64	10	4.46	–				
	R	10	64	10	3.28					
Other	–					–				

Negative correlations between brain signal and participants’ mean *importance* ratings were also detected, but only for self traits: bilaterally in orbitofrontal cortex, basal forebrain, medial prefrontal cortex, anterior cingulate cortex, paracentral gyrus, posteromedial cortex, caudate, putamen, insula and superior temporal gyrus, and in the left postcentral gyrus (Table 6 and Table S7).

**Table 6:** Activity in CMSs **negatively** correlated with participants’ importance ratings. Coordinates (*x*, *y*, *z*; MNI-152 standard space) and *Z*-scores correspond to the activation peaks (clusters *Z* > 2.3; cluster probability *p* < .05) in the MPFC and PMC. (See Tables S5 and S6 for the remaining whole-brain results.)

	**MPFC**					**PMC**				
	H	*x*	*y*	*z*	*Z*	H	*x*	*y*	*z*	*Z*
**Facts**										
Self	–					–				
Other	–					–				
**Traits**										
Self	L	−2	34	−10	3.54	–				
	R	4	38	−14	3.61					
Other						–				

#### Amount of memory retrieved to answer the questions

The participants’ estimates of the amount of memory retrieved to answer the questions correlated positively with signal in three conditions (Table 5 and Table S8): (i) for self-facts, in the left precuneus, postcentral gyrus and lateral occipital lobe, and in the right inferior frontal, superior temporal, middle temporal, and supramarginal gyri; (ii) for self-traits, in the right putamen, thalamus, internal and external capsule, and bilaterally in the medial prefrontal region, anterior cingulate cortex, middle frontal gyrus, orbitofrontal region and frontal pole; (iii) for other-facts, in the left paracentral gyrus, precuneus, postcentral gyrus and superior parietal lobule.

## Discussion

Our results show that, both for self and other, evaluative tasks regarding the two domains of information generated greater activity in CMSs compared with baseline. Moreover, the four experimental conditions engaged regions related to semantic memory (e.g., Binder et al., 2009), autobiographical and episodic memory (e.g., Cabeza & St Jacques, 2007), and somatic representations related to emotion and decision-making (e.g., Nakao, Ohira & Northoff, 2012). These results support the notion that CMSs activity during evaluative tasks of biographical information relates to processes of memory retrieval and decision underlying the tasks, for both self and other. In the paragraphs below, we discuss the relevant findings with respect to autobiographical-self processes, the distinction of self versus other, and the role of cortical midline structures.

### Autobiographical self

An autobiographical-self state requires access to sets of memories that are stored over a lifetime of experiences. Once retrieved, such memories may trigger emotional reactions and produce further retrieval of related memories. Consequently, an autobiographical-self state may range from relatively simple, as when one displays landmark facts relative to one’s date and place of birth, to fairly more complex, as when one is asked to describe a specific event or period of one’s life (Damasio, 1998). Our results support these assumptions. As noted earlier, however, the experimental task appears to require both memory retrieval and decision processes, indicating that the latter needs also to be considered.

#### Differences between biographic facts and personality traits

The data we obtained demonstrate that evaluating autobiographic information varies according to the domain of information. The response times to the questions were shorter for self-facts than for self-traits. This advantage suggests that both memory and decision processes are more straightforward for facts than for traits. As explained before, given the relevance of facts regarding one’s identity in daily life, it is possible that individuals hold less ambiguous and more objective, summarized, memory representations for biographic facts than for traits. This possibility is supported by our results showing that mean descriptive ambivalence scores were lower for facts than for traits.

Compared with self-traits, self-facts were associated with greater activity in the PMC, MPFC, hippocampus and basal forebrain. This is probably due to the processes of retrieval given the role of these structures in memory (e.g., Binder et al., 2009) and may indicate that questions about facts triggered a greater amount of memory retrieval than the questions about traits. We note that the questions were presented for 4 s in blocks of 24 s and thus this greater activity might even be due to retrieval of additional memories occurring *after* the questions are answered rather than to the retrieval needed to answer the questions in the first place. In any case, as discussed above, the number of daily events and experiences regarding one’s facts is probably greater than that regarding one’s traits, and thus an individual is likely to hold a greater amount of memories for facts than for traits. Nonetheless, it is possible that this activity relates not only to the amount of memory retrieved but also the kind of the memories retrieved, such as the age of those memories. Specifically, because facts are especially relevant to one’s everyday life, they may be preferentially associated with recent memories. We know that recent memories compared with past memories are associated with greater activity in the PMC (D’Argembeau et al., 2008; Gilboa, 2004; Söderlund et al., 2011) and in the MPFC (D’Argembeau et al., 2008).

Self-traits were associated with greater activity in the anterior insular cortices compared with self-facts. Given that the role of the insular cortices in somatic representations and in feeling states is well established (reviewed in Damasio & Carvalho, 2013), our finding suggests that, compared with facts, traits elicited more emotion-related representations. These representations may occur as responses to the memories retrieved or as part of processes of decision-making, such as error monitoring and conflict solving during decision-making (Orr & Hester, 2012). Furthermore, these responses are likely to be greater for traits than for facts because of the greater emotional significance of traits. Traits vary in valence and desirability (Anderson, 1968), and individuals seem to prefer to be associated with socially desirable traits, as our data on descriptiveness demonstrate and other authors have also noted (Klein & Loftus, 1993). Furthermore, as indicated above, the representations for traits are more likely to be ambivalent than those for biographical facts and thus traits, compared with facts, may be associated with a less straightforward decision-making process. Nonetheless, we note that memories retrieved for facts are also likely to evoke emotional responses. Our results showed that self-facts compared with self-traits yielded greater activity in the left amygdala, which may well relate to emotional responses to the memories retrieved given that the amygdala has been associated with the retrieval of memories with high emotional content (Denkova, Dolcos & Dolcos, 2013; McGaugh, 2013).

#### Differences across individuals

The level of brain activity varied across individuals with regard to the three variables that we investigated. Specifically, the level of brain activity in relevant structures such as the PMC and the MPFC correlated with participants’ descriptive ambivalence scores (i.e., how far the participants’ ratings of descriptiveness were from fully descriptive or from fully non-descriptive) and with participants’ importance scores. In other words, the level of activity in CMSs seems to be lower for individuals who considered the information unambiguously descriptive or non-descriptive and are thus likely to have required lower level of processing related to memory retrieval and decision making. Activity in the MPFC was lower for individuals who considered the traits more important to their self-image than for individuals who considered the traits less important in the same regard, suggesting that individuals who have given prior consideration to the information require lower level of processing related to memory retrieval and decision making to answer the questions. Those individuals also showed less activity for traits in structures related to emotional processing such as anterior cingulate and the insula, which seems to support the notion that traits are associated with emotional reactions.

Our findings regarding descriptiveness ambivalence and importance are in line with findings from another study, which has shown that level of activity in the MPFC and insula correlated positively with the level of difficulty associated with decision-making (Meffert et al., 2013). In addition, these results do not seem to support the notion that activity in the MPFC is commensurate with the level of descriptiveness and importance of self-related information evaluated, proposed by other studies (D’Argembeau et al., 2012; Moran et al., 2006). We note that there are, however, several differences between those studies and our current study, which limit the possibility of comparing results across the studies.

Furthermore, participants who reported having retrieved a greater amount of memory to answer the questions seemed to require greater levels of activity in the structures related to memory retrieval, such as the PMC. Altogether, these results seem to support the notion that brain activity for evaluation of biographic information varies across individuals, depending on the level of processing related to memory retrieval and decision making required to answer the questions.

### Self versus other

Our data on the comparison of each condition with the baseline support the idea that self and other engage some of the same structures, including CMSs. This is consistent with the proposal that two processes required to evaluate biographic information–memory retrieval and decision-making–are comparable for self and for other. Similarly to what we observed for self, brain activity related to other appears to vary with the domain of information evaluated (traits versus facts), as well as with individual differences on descriptiveness (how well the information targeted by the questions described other), and estimates of amount of memory retrieval that was required to answer the questions.

The results also reveal intriguing differences between self and other. Compared with other, self was associated with shorter response times, smaller perceived amount of memory retrieved to answer the questions, and lower levels of activity in CMSs’ integrative hubs such as the PMC. These significant differences were probably influenced by our choice of other and were related to differences regarding the type and accessibility of the memories between self and a distant other. Autobiographical memories derive from a multitude of instants of self-knowledge distributed over a person’s lifetime. Consequently, the amount of autobiographical memories is very large, enabling individuals to abstract summary representations from some of those autobiographical memories. Those summary representations, in turn, probably allow individuals to answer self-related questions without needing to retrieve particular episodes or events (Klein & Loftus, 1993). On the other hand, as explained before, the representation of a distant acquaintance’s biography derives from events and experiences that may have occurred during limited interactions over the course of the acquaintanceship. Thus memories for a distant acquaintance are probably less numerous, less frequently retrieved, and less readily accessible than autobiographical memories. In addition, individuals are less likely to hold summary representations for a distant acquaintance (Fuhrman & Funder, 1995) and thus will require greater level of memory retrieval in order to answer questions related to that acquaintance. Our data on the descriptive ambivalence scores, and on importance ratings seem to support this view. We note, however, that individuals’ knowledge of other may be similar to that of self when the other is a close acquaintance (Fuhrman & Funder, 1995), possibly explaining discrepancies in the published results.

Unlike what was observed for self, other-traits compared with other-facts were not associated with greater insula activity. Considering the association of the insula with affective processing, this difference suggests that emotion-related somatic representations are more prominent for self than for a distant other. This finding too may be explained by our choice of other. Given the lack of a close relationship between the participants and the acquaintances, the emotional significance of the retrieved memories and of the related decision-making is possibly less for other than for self. Of note, although positive traits were considered more descriptive than negative traits for both self and other, the difference was greater for self than for other. Also, positive traits were considered more important than negative traits for self but not for other. A recent meta-analysis of studies of self-reference, many of which explored the domain of personality traits, also suggests that self is associated with greater activity in the insula than other (Qin et al., 2011).

### Cortical midline structures

Overall, the results support the notion that CMSs are involved in processes of self. Nonetheless, the involvement also occurs for other. Moreover, the level of activity in CMSs appears to increase for conditions requiring greater level of processing related to memory retrieval and decision making. Still, the data also suggest that there are functional differences for subcomponents of the CMSs, namely between the MPFC and the PMC.

#### MPFC

The MPFC is connected with structures involved in memory processing, such as the hippocampus, as well as with structures involved in somatic representations related to emotions, such as nuclei in the brainstem tegmentum, amygdala and cingulate cortex (reviewed in Fuster, 2008). It has been suggested that the MPFC is also engaged in the integration of emotion-related somatic signals in decision-making (Bechara, Tranel & Damasio, 2000). Our data seem to support this suggestion, by showing the activity in the MPFC correlated with descriptive ambivalence and importance, which are presumably commensurate with how straightforward the decision-making processes were. Data from a more recent study showed also that activity in the MPFC during traits correlated positively with the decision difficulty (Meffert et al., 2013).

It is possible that activity in the MPFC relates also to memory retrieval, particularly to processes of emotional and perceptual representations related to the retrieved memories. Such processes could contribute, for example, to a “felt-rightness” during memory retrieval, as proposed in Moscovitch & Winocur (2002). Our data regarding the difference between facts and traits support this idea, given that facts compared with traits are, as noted before, likely to be associated with greater amount of autobiographical memories and even greater amount of recent memories. Other results are in line with this view: (i) high-experience domains of autobiographic information compared with low-experience domains of autobiographical information engage greater activity in MPFC, nucleus accumbens and amygdala (Lieberman, Jarcho & Satpute, 2004); (ii) current autobiographical information compared with past autobiographical information involves greater activity in the MPFC (D’Argembeau et al., 2008); and (iii) activity in the MPFC correlates with the participants’ success in retrieving information that pertains to self (Macrae, 2004) and to non-self (Schnyer, Nicholls & Verfaellie, 2005).

We suspect that MPFC activity for other depends on the same factors, and thus the differences between self and other in terms of MPFC activity are likely to relate to emotional processing associated with decision-making and memory retrieval. Moreover, because the level of emotional processing tends to be greater for self than for other, it is possible that MPFC is generally more active for self than for other, as supported by a recent meta-analysis of studies comparing self-traits and other-traits (Araujo, Kaplan & Damasio, 2013). As indicated before, there is some evidence suggesting that those differences depend on who the other is in relation to self. For example, activity in the MPFC is not different for self and for a close friend, but it is greater for self than for a distant other (Ochsner et al., 2005). Along these same lines, the difference in MPFC activity between self and the participant’s mother has been shown to depend on the participants’ cultural background (Zhu et al., 2007). Intriguingly, we did not find a difference of MPFC activity between self and other in our study, raising the possibility that differences between self and other in terms of MPFC activity do not depend solely on the kind of affective relationship between self and other. Although the participants were not emotionally connected with their acquaintances, they knew them well enough to answer questions about their lives and personalities. Thus this finding may well suggest that individuals’ activity in the MPFC for another person depends also on how well they know that person.

#### PMC

The PMC has been shown to be a central hub of cortical connectivity (e.g., Hagmann et al., 2008; Parvizi et al., 2006). It seems to be engaged by integrative general-purpose processes (reviewed in Damasio, 2010), and its activity correlates with the state of awareness. For example, its activity increases gradually as coma lightens and moves toward vegetative stage and to full awareness (Laureys, Owen & Schiff, 2004). During the evaluation of biographic information, the PMC is likely to be particularly involved in retrieving and assembling memory fragments. Also, the PMC is severely compromised in Alzheimer’s disease, Wernicke-Korsakoff’s amnesia and post-anoxic amnesia (Laureys, Owen & Schiff, 2004), and is engaged by memory tasks, pertaining to autobiographical and non-autobiographical information (e.g., Binder et al., 2009). Furthermore, as noted above, PMC activity is greater for other compared with self, and for facts compared with traits. This is consistent with recent meta-analyses that show greater activity in PMC for other compared with self (e.g., Araujo, Kaplan & Damasio, 2013; Qin et al., 2011). In addition, PMC activity was smaller for participants who considered traits less ambivalent, and it was greater for participants who reported having retrieved greater amount of memories to answer the questions about facts; these findings support our view that the PMC’s involvement in memory is commensurate with memory effort, memory load or both. Data from other studies support this interpretation. The PMC has been shown to be more active (i) during recall of recent memories, either real or fictitious, than recall of recently *seen* objects (Hassabis, Kumaran & Maguire, 2007), (ii) during recall of information than repetition of information (Buckner et al., 1996; Schacter et al., 1996), (iii) during recall of a greater number of words than recall of a smaller number of words (Schacter et al., 1996), and (iv) during processing of recent memories than processing old memories (Gilboa, 2004; Söderlund et al., 2011).

## Conclusion

The level of activity in CMS during the performance of tasks designed to evaluate biographic information seem to depend on varied factors related to the memories elicited by the questions, and factors related to the processes of decision-making required to answer the questions. The evidence does not support the notion that autobiographical-self processes depend primarly or mostly on CMSs, or that the CMSs are specifically devoted to self-related processing. The results are compatible with the notion that CMSs are hubs capable of assisting different processes, ranging from those that occur during resting states (e.g., mind wandering), to those that underlie the evaluation of biographical information.

## Supplemental Information

10.7717/peerj.481/supp-1 Supplemental Information Supplemental Information. Tables reporting coordinates (*x*, *y*, *z*; MNI-152 standard space and) and *Z*-scores corresponding to the activation peaks in the whole brain for the analyses performed
