# Supplementary material for: Involvement of cortical midline structures in the processing of autobiographical information
Source: PeerJ. 2014 Jul 22;2:e481. doi: 10.7717/peerj.481 (PMC4121543; doi:10.7717/peerj.481)
Supplement: Supplemental Information [file peerj-02-481-s001.doc]

Supplementary Material

**Table S1.** Task versus baseline. Coordinates (x, y, z; MNI-152 standard space and) and Z-scores correspond to the activation peaks (clusters *Z* > 2.3; cluster probability *p* < .05) from a conjunction analysis of each condition minus baseline.

| **Structure** |  | **H** |  | **x** | **y** | **z** |  | **Z** |
| --- | --- | --- | --- | --- | --- | --- | --- | --- |
|  |  |  |  |  |  |  |  |  |
| Medial prefrontal cortex |  | L |  | -2 | 60 | -14 |  | 5.67 |
|  |  | R |  | 2 | 52 | -16 |  | 4.95 |
| Posteromedial cortex |  | L |  | -6 | -54 | 20 |  | 6.17 |
|  |  | R |  | 2 | -52 | 20 |  | 5.25 |
| Cuneus |  | L |  | -2 | -82 | 10 |  | 5.44 |
|  |  | R |  | 2 | -82 | 14 |  | 5.01 |
| Inferior frontal gyrus |  | L |  | -48 | 34 | -10 |  | 6.18 |
|  |  | R |  | 50 | 34 | -12 |  | 4.55 |
| Middle frontal gyrus |  | L |  | -44 | 12 | 54 |  | 4.20 |
| Superior frontal gyrus |  | L |  | -6 | 20 | 62 |  | 5.36 |
| Middle temporal gyrus |  | L |  | -50 | -38 | -4 |  | 5.93 |
|  |  | R |  | 56 | 8 | -28 |  | 3.93 |
| Temporal pole |  | L |  | -50 | 16 | -18 |  | 4.81 |
|  |  | R |  | 46 | 22 | -32 |  | 4 |
| Lateral occipital / angular gyrus |  | L |  | -50 | -64 | 28 |  | 5.19 |
| Basal forebrain/ orbitofrontal |  | L |  | -30 | 14 | -26 |  | 5.03 |
|  |  | R |  | 26 | 14 | -24 |  | 2.87 |
| Hippocampus |  | L |  | -20 | -28 | -8 |  | 5.49 |
|  |  | R |  | 24 | -26 | -8 |  | 5.11 |
| Amygdala |  | L |  | -16 | -6 | -18 |  | 3.43 |
| Cerebellum |  | L |  | -18 | -90 | -26 |  | 5.13 |
|  |  | R |  | 26 | -86 | -34 |  | 6.38 |
| Caudate |  | L |  | -10 | 8 | 10 |  | 4.73 |
| Thalamus |  | L |  | -4 | -14 | 8 |  | 4.22 |
|  |  | R |  | 6 | -10 | 0 |  | 3.22 |

**Table S2.** Self versus other. Coordinates (x, y, z; MNI-152 standard space and) and Z-scores correspond to the activation peaks (clusters *Z* > 2.3; cluster probability *p* < .05).

|  | | | | | | | | |
| --- | --- | --- | --- | --- | --- | --- | --- | --- |
| **Structure** |  | **H** |  | **x** | **y** | **z** |  | **Z** |
|  | | | | | | | | |
| **Self > other** | | | | | | | | |
| **Facts** | | | | | | | | |
| Postcentral gyrus |  | L |  | -66 | -18 | 22 |  | 2.97 |
| Supramarginal gyrus |  | L |  | -64 | -42 | 32 |  | 4.13 |
|  |  | R |  | 66 | -38 | 42 |  | 3.83 |
| Angular gyrus |  | L |  | -58 | -54 | 16 |  | 2.97 |
|  |  | R |  | 66 | -48 | 34 |  | 3.66 |
| Superior temporal gyrus |  | L |  | -58 | -54 | 16 |  | 2.97 |
|  |  | R |  | 66 | -28 | 20 |  | 2.66 |
| Middle temporal gyrus |  | R |  | 64 | -46 | 4 |  | 3.52 |
|  | | | | | | | | |
| **Other > self** | | | | | | | | |
| **Facts** | | | | | | | | |
| Posteromedial cortex |  | L/R |  | 0 | -70 | 26 |  | 4.22 |
|  |  | L |  | -8 | -48 | 0 |  | 3.62 |
| Posteromedial cortex |  | R |  | 4 | -54 | 18 |  | 3.78 |
|  | | | | | | | | |
| **Traits** | | | | | | | | |
| Posteromedial cortex |  | L |  | -2 | -68 | 22 |  | 4.59 |
|  |  | R |  | 6 | -56 | 18 |  | 5.36 |
| Lateral occipital cortex |  | L |  | -42 | -66 | 52 |  | 3.46 |
|  | | | | | | | | |
| **Facts and traits combined** | | | | | | | | |
| Posteromedial cortex |  | L |  | -8 | -48 | 2 |  | 4.77 |
|  |  | R |  | 6 | -56 | 18 |  | 5.8 |

**Table S3.** Self-facts versus self-traits. Coordinates (x, y, z; MNI-152 standard space and) and Z-scores correspond to the activation peaks (clusters *Z* > 2.3; cluster probability *p* < .05).

| **Structure** | |  | **H** |  | **x** | **y** | **z** | **Z** |
| --- | --- | --- | --- | --- | --- | --- | --- | --- |
|  |  |  | | | | | | |
| **Facts > traits** | | | | | | | | |
| Medial prefrontal cortex | |  | L |  | -10 | 26 | -16 | 4.92 |
|  | |  | R |  | 8 | 30 | -18 | 5.06 |
| Posteromedial cortex | |  | L |  | -4 | -64 | 20 | 6.04 |
|  | |  | R |  | 2 | -58 | 20 | 5.98 |
| Middle frontal gyrus | |  | L |  | -24 | 18 | 44 | 5.7 |
|  | |  | R |  | 28 | 18 | 48 | 5.83 |
| Precentral gyrus | |  | L |  | -42 | 0 | 30 | 4.13 |
|  | |  | R |  | 50 | 4 | 8 | 2.99 |
| Middle temporal gyrus | |  | L |  | -56 | -36 | -12 | 5.24 |
|  | |  | R |  | 64 | -38 | -6 | 4.54 |
| Inferior temporal gyrus | |  | L |  | -62 | -52 | -12 | 5.12 |
|  | |  | R |  | 58 | -42 | -12 | 4.96 |
| Angular gyrus | |  | L |  | -50 | -56 | 40 | 4.9 |
|  | |  | R |  | 60 | -54 | 30 | 5.2 |
| Supramarginal gyrus | |  | L |  | -56 | -48 | 36 | 3.98 |
| Lateral occipital | |  | L |  | -40 | -76 | 42 | 6.72 |
|  | |  | R |  | 48 | -70 | 32 | 6.83 |
| Fusiform gyrus | |  | L |  | -30 | -38 | -18 | 5.5 |
|  | |  | R |  | 26 | -38 | -18 | 3.52 |
| Hippocampus | |  | L |  | 28 | -24 | -28 | 3.69 |
|  | |  | R |  | -26 | -14 | -26 | 3.52 |
| Amygdala | |  | L |  | -14 | -6 | -16 | 2.51 |
| Basal forebrain | |  | L |  | -8 | -6 | -16 | 2.51 |
|  | |  | R |  | 8 | 6 | -16 | 3.15 |
|  | | | | | | | | |
| **Traits > facts** | | | | | | | | |
| Inferior frontal gyrus | |  | L |  | -54 | 22 | -6 | 4.26 |
| Insula | |  | L |  | -40 | 14 | -12 | 3.01 |
| Orbitofrontal | |  | L |  | -42 | 20 | -14 | 3.16 |
| Lateral occipital | |  | R |  | 42 | -92 | 8 | 4.71 |
|  |  |  | | | | | | |

**Table S4.** Other-facts compared with other-traits. Coordinates (x, y, z; MNI-152 standard space and) and Z-scores correspond to the activation peaks (clusters *Z* > 2.3; cluster probability *p* < .05) for other-facts > other-traits. The reverse contrast did not yield statistical significant result in this analysis.

| **Structure** |  |  | **x** | **y** | **z** |  | **Z** |
| --- | --- | --- | --- | --- | --- | --- | --- |
|  |  |  |  |  |  |  |  |
| Medial prefrontal cortex | L |  | -8 | 32 | -16 |  | 4.3 |
|  | R |  | 10 | 36 | -18 |  | 3.88 |
| Posteromedial cortex | L |  | -4 | -60 | 16 |  | 5.79 |
|  | R |  | 2 | -54 | 12 |  | 5.01 |
| Middle frontal gyrus | L |  | -24 | 14 | 48 |  | 5.44 |
|  | R |  | 28 | 18 | 54 |  | 4.9 |
| Precentral gyrus | L |  | -38 | -4 | 56 |  | 4.24 |
| Cuneus | L |  | -6 | -66 | 4 |  | 4.71 |
|  | R |  | 6 | -76 | 2 |  | 4.73 |
| Middle temporal gyrus | L |  | -60 | -14 | -14 |  | 4.92 |
|  | R |  | 60 | -32 | -10 |  | 3.6 |
| Inferior temporal gyrus | L |  | -62 | -52 | -14 |  | 5.41 |
|  | R |  | 60 | -46 | -14 |  | 4.5 |
| Angular gyrus | L |  | -50 | -56 | 50 |  | 4.5 |
|  | R |  | 50 | -84 | 44 |  | 3.23 |
| Supramarginal gyrus | L |  | -52 | -52 | 20 |  | 4.07 |
| Lateral occipital | L |  | -36 | -72 | 40 |  | 5.53 |
|  | R |  | 48 | -70 | 34 |  | 6.16 |
| Fusiform gyrus | L |  | -30 | -38 | -18 |  | 5.02 |
|  | R |  | 32 | -28 | -24 |  | 3.7 |
| Hippocampus | L |  | -22 | -12 | -26 |  | 4.52 |
|  | R |  | -20 | -12 | -30 |  | 4.21 |
| Amygdala | L |  | -18 | -8 | -16 |  | 4.21 |
| Cerebellum | L |  | -12 | -74 | -28 |  | 4.8 |
|  | R |  | 8 | 74 | -28 |  | 4.33 |
| Thalamus | L |  | -16 | -30 | -2 |  | 2.93 |
| Pons | L |  | -4 | -32 | -32 |  | 3.81 |
| Medulla | L |  | -2 | -40 | -46 |  | 2.8 |

**Table S5. Brain activity and descriptive ambivalence scores for self. Coordinates (x, y, z; MNI-152 standard space and) and Z-scores correspond to the activation peaks (clusters *Z* > 2.3; cluster probability *p* < .05) positively correlated with participants’ mean descriptive ambivalence for each condition.**

|  | | | | | | | | |
| --- | --- | --- | --- | --- | --- | --- | --- | --- |
| **Structure** |  | **H** |  | **x** | **y** | **z** |  | **Z** |
|  | | | | | | | | |
| **Self- facts** | | | | | | | | |
| Medial prefrontal cortex |  | L |  | -10 | 38 | 34 |  | 3.75 |
| Medial prefrontal cortex/  Anterior cingulate cortex |  | L |  | -6 | 48 | 6 |  | 3.11 |
|  |  | R |  | 2 | 50 | 6 |  | 3.24 |
| Superior frontal gyrus/  Middle frontal gyrus |  | L |  | -18 | 40 | 30 |  | 3.35 |
|  |  | R |  | 14 | 38 | 40 |  | 3.79 |
| Cerebellum |  | L |  | -44 | -52 | -36 |  | 4.09 |
|  |  | R |  | 46 | -48 | -38 |  | 4.36 |
|  | | | | | | | | |
| **Self-traits** | | | | | | | | |
| Paracentral gyrus |  | L |  | -4 | -34 | 70 |  | 3.15 |
|  |  | R |  | 6 | -36 | 68 |  | 2.31 |
| Posteromedial cortex |  | L |  | -4 | -78 | 46 |  | 3.47 |
|  |  | R |  | 6 | -52 | 62 |  | 2.8 |
| Middle frontal gyrus |  | R |  | 40 | 52 | 24 |  | 4.13 |
| Precentral gyrus/ postcentral gyrus/  Superior parietal lobule |  | L |  | -34 | -30 | 50 |  | 4.88 |
|  |  | R |  | 38 | -34 | 46 |  | 3.99 |
| Superior parietal lobule |  | R |  | 48 | -52 | 30 |  | 3.57 |
| Mesencephalon |  | L |  | -2 | -24 | -20 |  | 3.89 |
| Pons |  | R/L |  | 0 | -20 | -32 |  | 3.65 |
| Paracentral gyrus |  | L |  | -4 | -34 | 70 |  | 3.15 |
|  | | | | | | | | |

**Table S6. Brain activity and descriptive ambivalence for other-traits.  Coordinates (x, y, z; MNI-152 standard space and) and Z-scores correspond to the activation peaks (clusters *Z* > 2.3; cluster probability *p* < .05) positively correlated with participants’ mean descriptive ambivalence for other-traits. No statistically significant correlations were found for other-facts.**

|  | | | | | | | | |
| --- | --- | --- | --- | --- | --- | --- | --- | --- |
| **Structure** |  | **H** |  | **x** | **y** | **z** |  | **Z** |
|  | | | | | | | | |
|  |  |  |  |  |  |  |  |  |
| **Other-traits** | | | | | | | | |
|  |  |  |  |  |  |  |  |  |
| Frontal pole |  | R |  | 14 | 54 | -10 |  | 3.81 |
| Orbitofrontal cortex |  | R |  | 8 | 30 | -24 |  | 3.57 |
| Medial prefrontal cortex |  | L |  | -2 | 32 | -20 |  | 2.66 |
|  |  | R |  | 2 | 32 | -24 |  | 3.95 |
| Posteromedial cortex |  | L |  | -8 | -70 | 54 |  | 3.17 |
|  |  | R |  | 14 | -74 | 56 |  | 3.86 |
| Cerebellum |  | L |  | -2 | -66 | -10 |  | 3.77 |
|  |  | R |  | 2 | -74 | -14 |  | 3.83 |
|  |  |  |  |  |  |  |  |  |
| Postcentral gyrus/  superior parietal lobule |  | L |  | -42 | -48 | 58 |  | 3.56 |
|  | | | | | | | | |

**Table S7. Brain activity and importance ratings for self-traits.  Coordinates (x, y, z; MNI-152 standard space and) and Z-scores correspond to the activation peaks (clusters *Z* > 2.3; cluster probability *p* < .05) negatively correlated with participants’ mean importance ratings for self-traits.**

|  | | | | | | | | |
| --- | --- | --- | --- | --- | --- | --- | --- | --- |
| **Structure** |  | **H** |  | **x** | **y** | **z** |  | **Z** |
|  | | | | | | | | |
|  |  |  |  |  |  |  |  |  |
| Orbitofrontal cortex/  Basal forebrain |  | R |  | 18 | 30 | -22 |  | 3.11 |
|  |  | L |  | -22 | 22 | -20 |  | 4.23 |
| Medial prefrontal cortex/  Anterior cingulate cortex |  | R |  | 4 | 38 | -14 |  | 3.61 |
|  |  | L |  | -2 | 34 | -10 |  | 3.54 |
| Paracentral gyrus |  | R |  | 6 | -40 | 68 |  | 3.61 |
|  |  | L |  | -2 | -36 | 70 |  | 4.62 |
| Posteromedial cortex |  | R |  | -4 | -50 | 64 |  | 3.65 |
|  |  | L |  | 6 | -52 | 62 |  | 3.29 |
| Caudate/ putamen |  | R |  | 6 | 12 | -8 |  | 4.1 |
|  |  | L |  | -16 | 16 | -12 |  | 4.79 |
| Postcentral gyrus/  Superior parietal lobule |  | L |  | -64 | -28 | 14 |  | 3.85 |
| Superior temporal gyrus |  | L |  | -58 | -6 | 2 |  | 3.25 |
| Insula/ superior temporal gyrus |  | R |  | 48 | -22 | 10 |  | 3.85 |
| Insula |  | L |  | -36 | -18 | 2 |  | 3.0 |

**Table S8.** Brain activity and memory retrieval estimates ratings for self and other.  Coordinates (x, y, z; MNI-152 standard space and) and Z-scores correspond to the activation peaks (clusters *Z* > 2.3; cluster probability *p* < .05) **positively** correlated with participants’ memory estimates for each condition.

|  |  |  | |  |  | |  |  |  |  |
| --- | --- | --- | --- | --- | --- | --- | --- | --- | --- | --- |
| **Structure** |  | **H** | |  | **x** | | **y** | **z** |  | **Z** |
|  | | | | | | | | | | |
| **Self** | | | | | | | | | | |
|  | | | | | | | | | | |
| **Facts** | | | | | | | | | | |
|  |  |  | |  |  | |  |  |  |  |
| Posteromedial cortex |  | L | |  | -6 | | -48 | 54 |  | 3.35 |
|  |  | L | |  | -20 | | -44 | 66 |  | 3.93 |
| Lateral occipital cortex |  | L | |  | -12 | | -68 | 54 |  | 3.69 |
|  |  | L | |  | -16 | | -68 | 52 |  | 3.66 |
| Inferior frontal gyrus |  | R | |  | 66 | | -14 | 12 |  | 4.16 |
| Superior temporal gyrus |  | R | |  | 66 | | -30 | 16 |  | 3.8 |
| Superior temporal gyrus*/*  Middle temporal gyrus |  | R | |  | 52 | | -22 | 6 |  | 3.2 |
| Supramarginal gyrus |  | R | |  | 46 | | -40 | 26 |  | 3.32 |
|  |  | |  | | |  | | | | |
| **Traits** | | | | | | | | | | |
| Medial prefrontal cortex |  | L | |  | -10 | | 64 | 10 |  | 4.46 |
|  |  | R | |  | 10 | | 64 | 10 |  | 3.28 |
| Anterior cingulate cortex /  Medial prefrontal cortex |  | L | |  | -4 | | 36 | 24 |  | 2.79 |
|  |  | R | |  | 4 | | 30 | 34 |  | 3.71 |
| Middle frontal gyrus |  | L | |  | -20 | | 40 | 40 |  | 4.71 |
|  |  | R | |  | 42 | | 38 | 26 |  | 3.83 |
| Frontal pole/  Orbitofrontal cortex |  | L | |  | -18 | | 52 | -14 |  | 2.46 |
|  |  | R | |  | 28 | | 46 | -14 |  | 4.54 |
| Orbitofrontal cortex |  | R | |  | 28 | | 54 | -18 |  | 3.7 |
| Putamen |  | R | |  | 24 | | 2 | 0 |  | 3.25 |
| Thalamus |  | R | |  | 18 | | -20 | 8 |  | 2.56 |
| Internal capsule |  | R | |  | 16 | | 0 | 14 |  | 3.96 |
| External capsule |  | R | |  | 26 | | 16 | -8 |  | 3.21 |
|  |  | |  | | |  | | | | |
| **Other** | | | | | | | | | | |
| **Facts** | | | | | | | | | | |
|  |  | |  | | |  | | | | |
| Paracentral gyrus |  | L | |  | -4 | | -24 | 76 |  | 4.03 |
| Posteromedial cortex |  | L | |  | -4 | | -46 | 60 |  | 3.31 |
| Postcentral gyrus |  | L | |  | -14 | | -44 | 68 |  | 3.55 |
| Superior parietal lobule |  | L | |  | -18 | | -50 | 68 |  | 3.8 |
